# Safety and pharmacokinetics of CL-197, a potent compound with low cytotoxicity against HIV-1: a randomized, double-blind, placebo-controlled, first-in-human phase 1 clinical trial

**DOI:** 10.1128/aac.00984-26

**Published:** 2026-09-04

**Authors:** Yuming Guo, Yonggang Li, Pan Zhao, Jingfeng Bi, Chaoyun Gou, Zhengran Chuai, Shuangjie Yu, Renjie Zhang, Jinfa Du, Yi Ou, Ruonan Xu, Na Guo, Fu-Sheng Wang, Yuanyuan Li

**Affiliations:** 1 Senior Department of Infectious Diseases, the Fifth Medical Center of Chinese PLA General Hospital, National Clinical Research Center for Infectious Diseases 26460, Beijing, China; 2 School of Traditional Chinese Medicine, Southern Medical University 70570, Guangzhou, Guangdong, China; 3 China-Japan Friendship Hospital 36635, Beijing, China; 4 Henan Genuine Biotech Co., Ltd., Pingdingshan, Henan, China; Chinese Academy of Medical Sciences & Peking Union Medical College, Beijing, China

**Keywords:** HIV, CL-197, pharmacokinetics, phase 1 clinical trial

## Abstract

HIV infection remains a critical global public health concern. A potential long-acting therapeutic agent, 4′-ethynyl-2-fluoroadenosine analog 1c (CL-197), was investigated in a first-in-human phase 1 clinical trial. This single-center, randomized, double-blind, dose-escalation, placebo-controlled study assessed the safety and pharmacokinetics (PKs) of five doses of CL-197 (1, 10, 30, 60, and 100 mg) in healthy adults aged 18–45 years. The 1 mg group was open-label (*n* = 4), while the other groups were randomized 4:1 to CL-197 or placebo. The primary endpoints were safety and tolerability. The PK evaluation involved the characterization of systemic exposure parameters (based on plasma drug concentrations and peripheral blood mononuclear cell [PBMC] drug concentrations) and urinary excretion parameters. CL-197 was well tolerated at all dose levels. The most frequently reported adverse reactions were sinus arrhythmia, followed by decreased apolipoprotein B, decreased blood iron, increased triglycerides, and elevated low-density lipoprotein. Plasma PKs showed dose-proportional exposure, with a mean time to maximum plasma concentration (*T*_max_) of 0.50–1.00 h. As the dose increased, the elimination time was prolonged. The plasma drug concentrations were approximately dose-proportional, whereas *AUC*_0-t_ and *AUC*_0-∞_ exhibited non-linear characteristics within the range of 1-100mg, with the mean elimination half-life (*t*_1/2_) increasing from 1.39 to 10.20 h across doses. The mean *t*_1/2_ in PBMCs was 61.29 h and 43.80 h in the 30 mg and 60 mg dose groups, respectively, and the median *T*_max_ was 12 h. In conclusion, a single administration of CL-197 demonstrated an acceptable safety profile in younger adults. The intracellular CL-197-TP in PBMCs had a long half-life, providing preliminary support for the potential use of extended dosing intervals in further development.

**CLINICAL TRIALS:**

This study is registered with ClinicalTrials.gov as NCT05944848.

## INTRODUCTION

HIV infection remains a critical global public health challenge. According to estimates from the Joint United Nations Programme on HIV/AIDS (UNAIDS), approximately 41 million people were living with HIV globally by the end of 2025, with 1.2 million new HIV infections occurring that year and 570,000 people succumbing to AIDS-related complications. Concurrently, 32.1 million individuals were receiving antiretroviral therapy (ART) (1, 2).

Scientific breakthroughs, the widespread availability of ART, and effective prevention methods have led to a substantial decline in both new HIV infections and HIV-related deaths (3). Although more than 30 anti-HIV drugs covering nucleoside reverse transcriptase inhibitors (NRTIs), non-nucleoside reverse transcriptase inhibitors (NNRTIs), protease inhibitors (PIs), integrase inhibitors (INSTIs), fusion inhibitors, and CCR5 antagonists have been approved for clinical use (4), low-level viral replication and the development of viral resistance continue to be major problems (5–7). There is an ongoing need for novel anti-HIV agents. This remains a major challenge for scientists and healthcare professionals worldwide.

Nucleoside analogs (NAs) represent a well-established class of compounds in medicinal chemistry and chemical biology. Among these, NRTIs were the first-in-class agents utilized extensively for HIV treatment (8–11). A significant subclass within NAs is the 4′-substituted nucleoside analogs, which play a vital role in antiviral drug discovery (12–14). Several modified derivatives, including azvudine (FNC), islatravir, censavudine, balapiravir, and AL-335, have advanced to clinical development.

2′-Deoxy-2′-β-fluoroadenosines bearing 4′-azido or 4′-ethynyl groups designed for the treatment of HIV-1 infection have been synthesized. These compounds possess nanomolar anti-HIV-1 activity, with the 4′-ethynyl-2-fluoroadenosine analog 1c (CL-197) being the most potent compound with low cytotoxicity (15). It has a similar mechanism of action to lamivudine (3TC), exhibits lower cytotoxicity toward cells, and demonstrates superior *in vitro* anti-HIV-1 activity compared to 3TC. CL-197 acts before the virus integrates into the host genome, effectively inhibiting the initiation of HIV-1 reverse transcription and the overall reverse transcription process of HIV-1. CL-197 also shows potent inhibitory activity against drug-resistant and clinical HIV-1 strains. The preclinical data support further development of CL-197 as a highly potent and orally bioavailable clinical candidate to treat HIV-1 infection. Moreover, it has the potential to be used as a long-acting antiretroviral (LA-ARV) drug. There are still relatively few LA-ARV drugs available for the treatment of HIV infection. Only three long-acting therapeutic options have been approved: cabotegravir plus rilpivirine, lenacapavir, and ibalizumab (16–20). Notably, no orally administered, long-acting antiretroviral agent has yet reached the market. Here, we report results from a phase 1 clinical trial evaluating the safety and pharmacokinetics (PKs) of CL-197 in healthy adults.

## MATERIALS AND METHODS

### Study design

**Fig 1:**
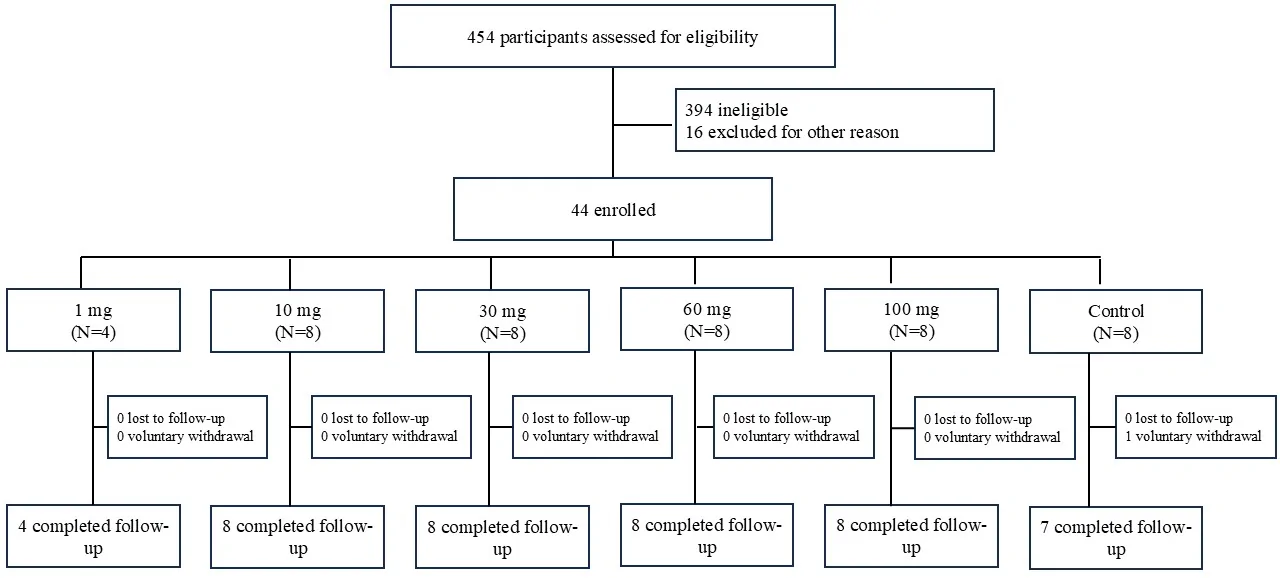
Trial profile: participant recruitment, randomization, and follow-up.

A single-center, randomized, double-blind, dose-escalation, placebo-controlled study was conducted to evaluate five doses of CL-197 (1 mg, 10 mg, 30 mg, 60 mg, and 100 mg) in adults without HIV. The first group (1 mg) was a single-center, one-arm, open-label trial in which all four participants received CL-197. Participants in the other dose groups received either CL-197 or a placebo, randomized at a ratio of 4:1 (Fig. 1). Blood routine, urine routine, blood biochemistry, electrocardiogram (ECG), blood lactate, and myocardial enzyme profile tests were conducted at 24 h, 4 days, 8 days, 15 days, 22 days, and 29 days after administration. Bone density and abdominal ultrasounds were performed 7 days after administration.

### Patient eligibility

For inclusion in the study, each participant was required to meet the following criteria: male or female, 18–45 years of age; body mass index (BMI) from 19 to 26 kg/m^2^, with a minimum weight of 50 kg for males and 45 kg for females; willingness to practice effective non-pharmacological contraceptive measures throughout the study period, with no pregnancy plans from 2 weeks prior to screening to 3 months after trial completion; and ability to comprehend and voluntarily sign the informed consent form.

Patients were not eligible to be included in the study if they met any of the following criteria: history of allergic predisposition or hypersensitivity to drugs/foods, particularly known allergies to any component of the investigational product or its excipients; documented history of hypoglycemia; clinically significant abnormalities in medical history, physical examination, laboratory tests, or study-related assessments, as determined by the investigator; regular smoking (≥5 cigarettes/day) within 12 months prior to screening; alcohol abuse (≥14 alcohol units/week; 1 unit = 285 mL beer, 25 mL spirits, or 150 mL wine) within 12 months prior to screening, or positive alcohol breath test (>0 mg/100 mL) at baseline; history of substance abuse within 12 months prior to screening, or positive test for addictive substances at baseline; major surgery within 3 months prior to screening that may affect drug absorption, distribution, metabolism, or excretion (ADME), or planned surgery during the study period; use of drugs with known interactions with the investigational product (e.g., strong CYP3A inhibitors: clarithromycin, indinavir, itraconazole) within 30 days prior to screening; history of clinically significant cardiovascular, hepatic, renal, pulmonary, gastrointestinal, or neurological disorders, or any condition potentially affecting drug ADME or posing risks to the subject; febrile illness within 7 days prior to screening; participation in other clinical trials within 3 months prior to screening; use of prescription/non-prescription drugs, herbal products, or dietary supplements within 14 days prior to screening; excessive daily consumption of tea, coffee, and/or caffeinated/xanthine-containing beverages (>8 cups/day; 1 cup = 250 mL) within 3 months prior to screening; blood loss or donation ≥200 mL within 8 weeks prior to screening; pregnant or lactating women; intolerance to venipuncture or history of needle/blood phobia; administration of COVID-19 vaccine within 1 week or other vaccines within 3 months prior to screening; or any other condition deemed ineligible by the investigator.

### Investigational products

The main component of CL-197 capsules is CL-197. It is a new type of long-acting nucleoside reverse transcriptase inhibitor with independent intellectual property rights owned by Henan True Biotechnology Co., Ltd., and it is intended for the treatment of HIV-1-infected individuals.

### Outcomes

The endpoints were the safety, tolerability, and PKs of CL-197. The number of participants with treatment-related adverse events (AEs) was assessed using Common Terminology Criteria for Adverse Events (CTCAE) version 5.0.

### Bioanalytical methods for CL-197

Plasma and urinary concentrations of CL-197 were measured using a validated liquid chromatography–tandem mass spectrometry bioanalytical method with multiple reaction monitoring and electrospray ionization (ESI) in positive mode. Mass spectrometry was performed in electrospray ionization (ESI) mode to monitor the precursor-to-product ion transitions (*m*/*z*) for the analyte CL-197 and the internal standard buspirone, which were 312.2 to 154 and 386.4 to 122.3, respectively. The bioanalytical method was validated over a calibrated range of 4.00–4,000.00 ng/mL.

CL-197-TP concentrations in peripheral blood mononuclear cells (PBMCs) were measured with a validated liquid chromatography–tandem mass spectrometry bioanalytical method with multiple reaction monitoring and electrospray ionization in the positive mode. A mass spectrometer was employed in ESI mode to monitor the parent ion and daughter ion mass-to-charge ratios (*m*/*z*) of the analyte CL-197-TP and the internal standard tolbutamide, which were 550.2 to 158.8 and 269.2 to 170.0, respectively. The bioanalytical method was validated over a calibrated range of 0.200–80.00 ng/mL. All samples were analyzed within the timeframe supported by frozen-storage stability data.

### Statistical analysis

The PK parameter analysis was conducted using Phoenix WinNonlin version 8.3.5 or higher versions for the estimation and analysis of non-compartmental model PK parameters. For the PK parameters, descriptive statistics were employed, including the number of analysis cases (*N*), number of missing cases (NMISS), number of BQLs (nBQL), mean (Mean), standard deviation (SD), median (Median), quartiles (*Q*_1_, *Q*_3_), minimum value (Min), maximum value (Max), coefficient of variation (CV%), and geometric mean (Geomean).

The non-PK parameters were analyzed using SAS version 9.4. For continuous variables, descriptive statistics were calculated, including the number of analysis cases (*N*), mean (Mean), standard deviation (SD), median (Median), minimum value (Min), and maximum value (Max). For categorical variables, the frequencies (*n*) and percentages were summarized.

## RESULTS

### Baseline characteristics of participants

A total of 454 participants were screened, of whom 410 were found to be ineligible or were excluded for other reasons. The most common reason for screen failure was not meeting the inclusion criteria. After exclusion, 44 participants were enrolled in the trial, including 31 males (70.45%) and 13 females (29.55%). One participant dropped out due to personal time constraints that rendered them unable to complete the trial according to the protocol. The first participant was enrolled on 27 July 2023, and the last was enrolled on 21 November 2023. The final study visit was completed on 20 January 2024. The demographics of the study population are shown in Table 1.

**TABLE 1:** Study participant demographics and baseline characteristics

	1 mg (*N* = 4) *n* (%)	10 mg (*N* = 8) *n* (%)	30 mg (*N* = 8) *n* (%)	60 mg (*N* = 8) *n* (%)	100 mg (*N* = 8) *n* (%)	Control (*N* = 8) *n* (%)
Gender, *n* (%)						
*N* (*N* Miss)	4 (0)	8 (0)	8 (0)	8 (0)	8 (0)	8 (0)
Male	3 (75.0)	4 (50.0)	6 (75.0)	6 (75.0)	6 (75.0)	6 (75.0)
Female	1 (25.0)	4 (50.0)	2 (25.0)	2 (25.0)	2 (25.0)	2 (25.0)
Age (years)						
*N* (*N* Miss)	4 (0)	8 (0)	8 (0)	8 (0)	8 (0)	8 (0)
Mean ± SD	32.75 ± 12.203	28.00 ± 7.783	31.25 ± 4.062	30.00 ± 5.043	30.63 ± 5.344	26.00 ± 5.345
Median (*Q*_1_, *Q*_3_)	34.00 (22.50, 43.00)	25.50 (22.50, 32.50)	30.50 (28.50, 33.50)	27.50 (26.50, 34.00)	31.50 (26.50, 34.50)	24.50 (21.50, 30.50)
Min–Max	19.0–44.0	20.0–43.0	26.0–39.0	25.0–39.0	22.0–38.0	21.0–34.0
Race, *n* (%)						
*N* (*N* Miss)	4 (0)	8 (0)	8 (0)	8 (0)	8 (0)	8 (0)
Han nationality	4 (100.0)	7 (87.5)	8 (100.0)	8 (100.0)	6 (75.0)	8 (100.0)
Other	0 (0.0)	1 (12.5)	0 (0.0)	0 (0.0)	2 (25.0)	0 (0.0)
BMI (kg/m^2^)						
*N* (*N* Miss)	4 (0)	8 (0)	8 (0)	8 (0)	8 (0)	8 (0)
Mean ± SD	22.93 ± 1.372	22.04 ± 1.883	21.80 ± 1.650	22.84 ± 1.784	23.85 ± 1.114	22.81 ± 1.762
Median (*Q*_1_, *Q*_3_)	22.65 (21.85, 24.00)	22.20 (20.20, 23.30)	21.55 (20.60, 22.30)	22.25 (21.90, 24.70)	24.10 (22.65, 24.75)	22.85 (21.70, 24.25)
Min–Max	21.7–24.7	19.9–25.0	20.2–25.3	20.0–25.0	22.5–25.3	19.8–25.1

### Safety and tolerability

The primary objective was to evaluate the safety and tolerability of CL-197 after single-dose administration. No serious adverse events (SAEs) or adverse events (AEs) leading to withdrawal were reported during this study. The most common AEs were grade 1, with a single grade 3 event (elevated blood creatine phosphokinase [CPK] in a placebo group), which was deemed unrelated to the study drug. The most frequently reported AEs were sinus arrhythmia, followed by decreased apolipoprotein B, elevated low-density lipoprotein cholesterol (LDL-C), decreased serum iron, elevated fasting triglycerides, abnormal T-waves on the electrocardiogram, sinus bradycardia, elevated apolipoprotein B, elevated lipoprotein(a) [Lp(a)], and elevated blood lactate (Table S1). AEs observed exclusively in the investigational drug group comprised abnormal T-waves on the ECG, sinus bradycardia, elevated Lp(a), and elevated blood lactate.

### Pharmacokinetics

The PK evaluation of CL-197 included the characterization of systemic exposure parameters (based on plasma drug concentrations and peripheral blood mononuclear cell [PBMC] drug concentrations) and urinary excretion parameters. An analysis of the PK parameters was performed for all participants who received CL-197.

**Fig 2:**
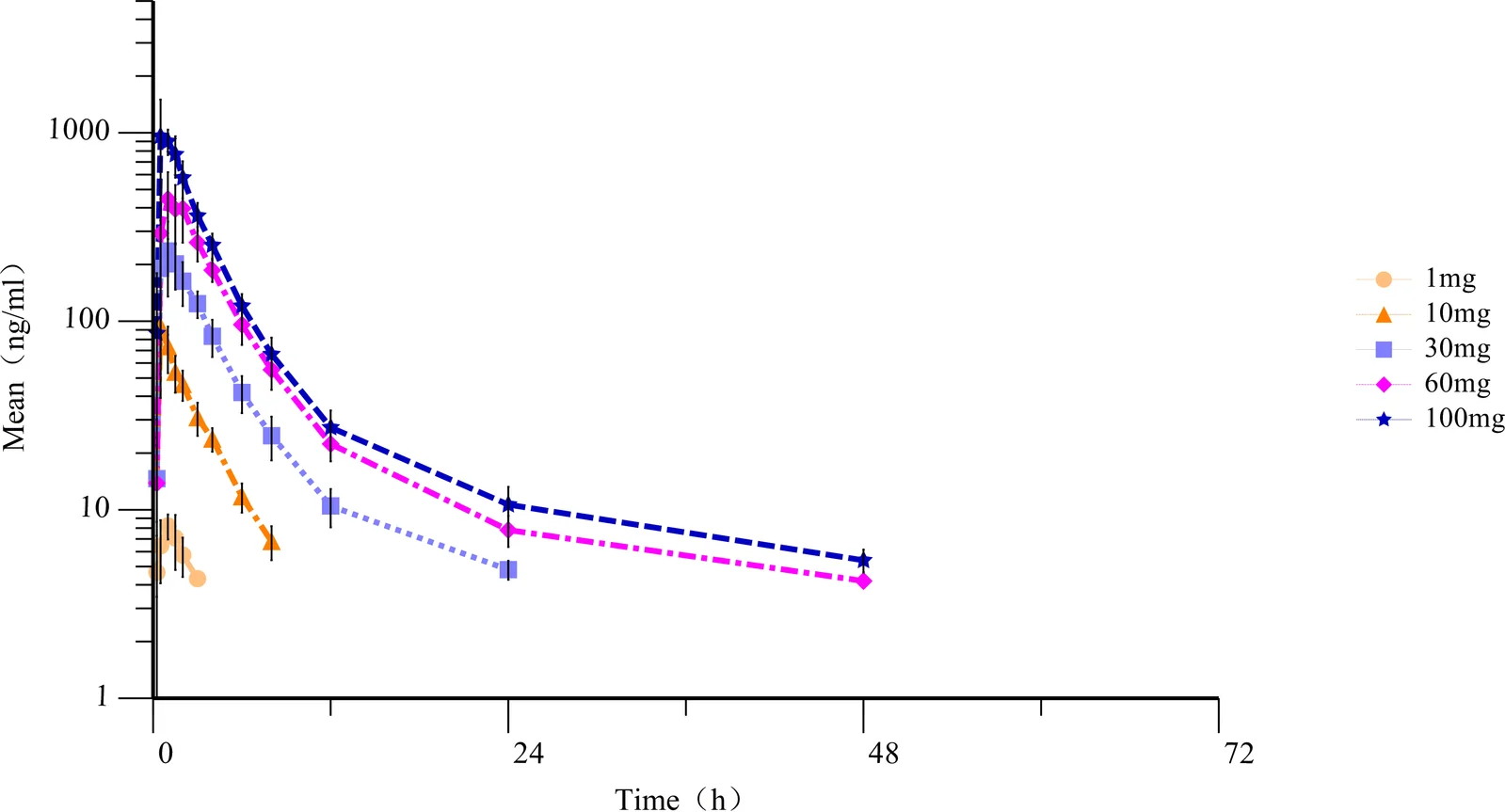
Mean plasma concentration-time profiles of CL-197.

The mean (±SD) maximum plasma concentration (*C*_max_) values following a single administration of CL-197 were 8.80 ± 1.663 ng/mL, 102.15 ± 31.472 ng/mL, 303.13 ± 92.303 ng/mL, 577.13 ± 102.754 ng/mL, and 1204.13 ± 257.136 ng/mL, respectively. The median time to maximum plasma concentration (*T*_max_) was 1.00 (0.50–1.50) h, 0.50 (0.50–1.00) h, 1.00 (0.50–2.00) h, 1.00 (0.50–2.00) h, and 0.50 (0.50–1.50) h, respectively. As the dose increased, the elimination half-life was prolonged. The median plasma elimination half-life (*t*_1/2_) was 1.39 (1.39–1.39) h, 2.20 (1.93–2.65) h, 4.10 (2.89–7.01) h, 5.61 (3.78–13.38) h, and 10.20 (3.35–15.40) h (Table S2). Plasma drug concentrations in subjects increased with escalating doses, indicating rapid absorption and extensive tissue distribution. As the dose increased, the drug elimination half-life was prolonged. This observation suggests a lack of dose proportionality in the primary PK parameters, consistent with non-linear PK characteristics within the tested dose range (Table S3). The concentration-time profiles for each dose group are detailed in Fig. 2.

**Fig 3:**
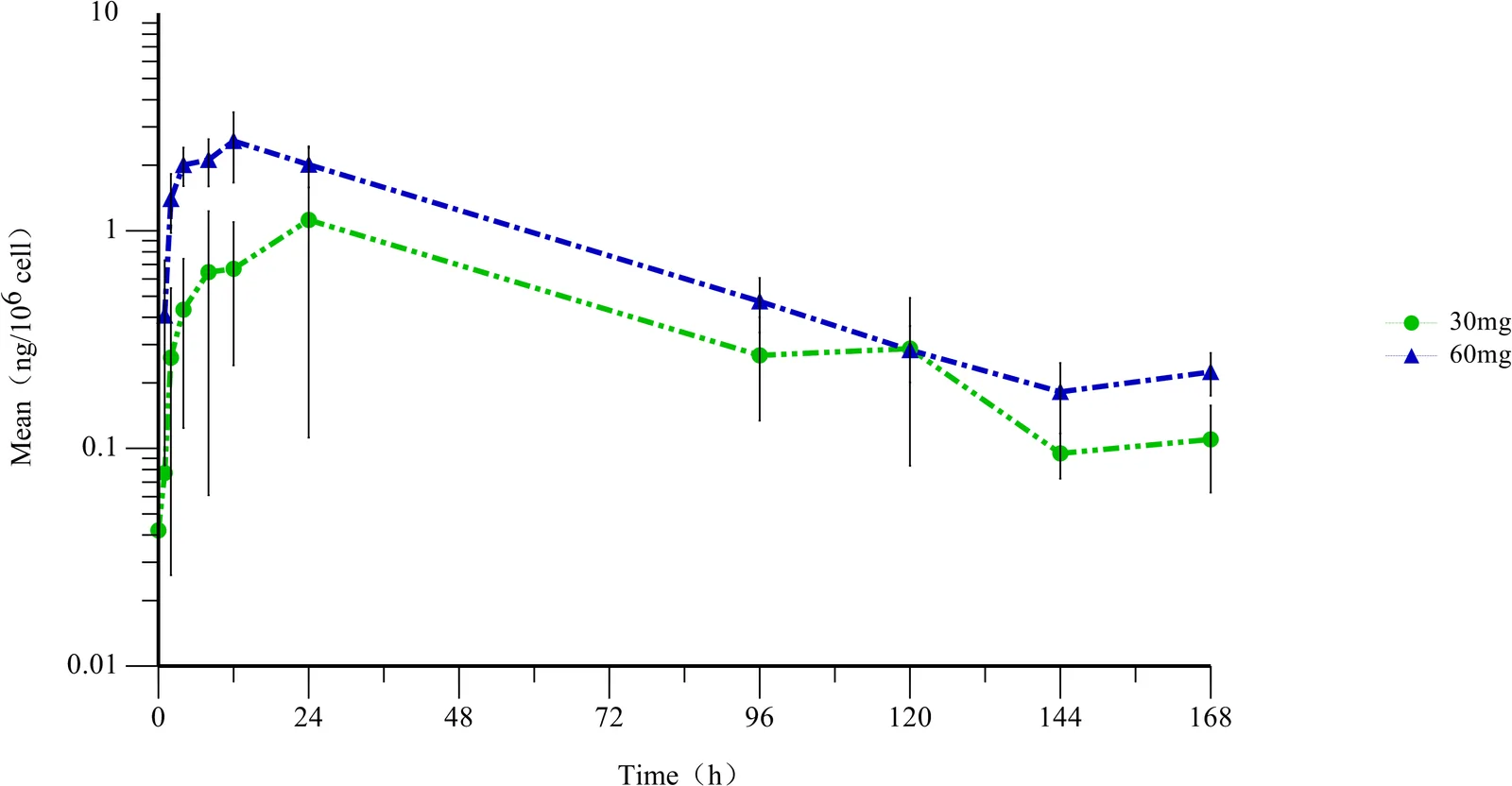
Mean CL-197-TP concentration-time profiles within PBMCs.

Subjects in the 30 mg and 60 mg dose groups underwent analysis of CL-197-TP concentrations in PBMCs and associated PK parameters. The mean (±SD) maximum concentration (*C*_max_) values were 1.34 ± 1.255 and 2.67 ± 0.835 ng/10^6^ cells, respectively, for these dose groups. The median time to maximum concentration (*T*_max_) was 12.00 (8.00–24.00) h and 12.00 (4.00–24.00) h. The median elimination half-life (*t*_1/2_) was 61.29 (19.40–78.67) h and 43.80 (39.65–99.94) h (Table S4). This indicates that a portion of the drug was distributed into PBMCs, achieving maximum concentration (*C*_max_) at 12 h post-dose. Within PBMCs, the drug was phosphorylated to its triphosphate metabolite, CL-197-TP, which exhibited slow elimination from these cells. The concentration-time profiles for each dose group are detailed in Fig. 3.

Urinary concentrations of CL-197 were analyzed in subjects across the 1–100 mg dose range. Cumulative urinary excretion from 0 to 72 h was 587.44 ± 64.01 µg, 6,388.03 ± 1,101.21 µg, 15,032.04 ± 3,140.40 µg, 32,005.39 ± 5,899.06 µg, and 65,498.38 ± 11,595.77 µg, respectively. The cumulative fraction recovered was 58.74% ± 6.40%, 63.88% ± 11.01%, 50.11% ± 10.47%, 53.34% ± 9.83%, and 65.50% ± 11.60%. These results indicate that the cumulative fraction recovered in urine over 0–72 h ranged from 50.11% to 65.50%, suggesting that >50% of the administered dose was excreted renally as unchanged drug within this timeframe. The urinary PK parameters are detailed in Table 2 and Table S5.

**TABLE 2:** Summary of cumulative urinary excretion and fraction excreted unchanged (mean ± SD, CV%)^*a*^

	1 mg (*N* = 4)	10 mg (*N* = 8)	30 mg (*N* = 7)	60 mg (*N* = 7)	100 mg (*N* = 8)
*A_e_*_0–72_ (µg)	587.44 ± 64.012 (10.9)	6,388.03 ± 1,101.205 (17.2)	15,032.04 ± 3,140.403 (20.9)	32,005.39 ± 5,899.056 (18.4)	65,498.38 ± 11,595.769 (17.7)
*F_e_*_0–72_ (%)	58.74 ± 6.399 (10.9)	63.88 ± 11.013 (17.2)	50.11 ± 10.469 (20.9)	53.34 ± 9.832 (18.4)	65.50 ± 11.596 (17.7)

^*a*^ Values with fraction excreted unchanged (*F_e_*) > 100% from two subjects were excluded from the data set.

## DISCUSSION

Lifelong ART is essential for individuals to achieve sustained viral suppression and immune reconstitution. However, the daily oral dosing regimen imposes a substantial treatment burden, often resulting in suboptimal adherence and significant psychological distress. Together, these factors increase the risk of virologic failure, viral evolution, and the emergence of drug resistance. Consequently, there is an urgent clinical need to develop LA-ARV formulations that reduce dosing frequency while maintaining therapeutic efficacy. LA-ARV can improve treatment adherence, enhance quality of life, and mitigate adherence-related anxiety associated with daily oral therapy. LA-ARV agents are anticipated to serve as either a valuable adjunct to or a potential replacement for conventional daily oral regimens.

Preclinical investigations suggest that CL-197 achieves sustained intracellular concentrations of its active metabolite 1c-TP in peripheral blood mononuclear cells (PBMCs) for several days (12). The PK characteristics in this study support the development of CL-197 as an oral long-acting regimen, potentially transforming HIV treatment by markedly improving adherence and patient quality of life.

Our data demonstrate that CL-197 was safe and well tolerated in healthy adults. No difference in AE incidence was observed across different dose levels. Following the single oral administration of different doses of CL-197 in healthy subjects, the observed abnormalities were primarily concentrated in the heart and related assessments, consistent with the effects observed in preclinical studies in dogs (12). Cardiac abnormalities mainly manifested as sinus bradycardia, sinus arrhythmia, and T-wave changes on electrocardiograms. No clear dose–response relationship was evident. However, the heart rates of the subjects with these abnormalities were either within the normal range or represented abnormalities without clinical significance. All abnormalities were mild in nature, classified as Common Terminology Criteria for Adverse Events (CTCAE) grade 1 in severity, resolved without intervention, and were potentially transient. Continued monitoring of these findings in subsequent clinical trials is warranted.

Abnormalities in laboratory parameters were primarily concentrated in biochemical indices, with the highest incidence observed in alterations related to lipid metabolism. All these abnormalities were mild in nature, classified as CTCAE grade 1 in severity, and resolved without intervention. Compared with similar drugs such as islatravir, CL-197 had similar safety and tolerability (21). It is important to note that the existing data cannot definitively establish a causal relationship between CL-197 and the observed lipid alterations, as lipid parameters are readily influenced by lifestyle and dietary factors. The potential effects of CL-197 on lipid metabolism should be further evaluated in subsequent studies.

PK evaluation of CL-197 in plasma across doses ranging from 1 to 100 mg suggested that *AUC _0-t_ and AUC _0-∞_* showed nonlinear pharmacokinetics, while the relationship between Cmax and dose was inconclusive regarding linearity. CL-197 was well absorbed, widely distributed, and had a short half-life. The half-life of CL-197-TP in PBMCs was significantly longer than that of CL-197 in plasma. CL-197-TP is the active form responsible for anti-HIV-1 activity. Its significantly longer half-life supports less frequent dosing.

The present study has several limitations. The highly selected, homogeneous, healthy volunteer population with the relatively narrow age range (18–45 years) limits the generalizability of the findings. While this design optimizes the internal validity of safety and PK assessments, it may not predict safety, tolerability, or PK parameters accurately in the intended patient population, who often have underlying disease states, compromised organ function, or concomitant medications that can profoundly influence drug disposition. This study represents the foundational step of a staged clinical program. Subsequent phase Ib/IIa studies will evaluate CL-197 in HIV-infected participants across a broader age range, including dedicated cohorts of older adults and those with impaired organ function.

While this study provides safety, tolerability, and PK data for single-dose administration, the combination of the single-dose design and small sample size may limit the ability to detect uncommon or rare AEs, potentially underestimating the true safety profile. Moreover, single-dose PK characterization cannot predict drug accumulation kinetics, time-dependent metabolic changes, or cumulative toxicity that may occur under steady-state conditions. Further evaluation of safety following multiple-dose administration is warranted. It will be necessary to characterize steady-state intracellular metabolite exposure, assess accumulation-related safety signals, and establish definitive dosing recommendations.

Finally, other practical factors, such as food effects and potential drug–drug interactions, were not evaluated due to protocol restrictions on diet and concomitant medications. Caution should be exercised when extrapolating these results, and confirmation in larger, longer-duration studies involving the target patient population is necessary.

### Conclusions

In this study, a single administration of CL-197 demonstrated an acceptable safety profile in younger adults.

The active metabolite CL-197-TP exhibited prolonged intracellular retention in PBMCs with a long elimination half-life, supporting the feasibility of extended dosing intervals in future clinical development. These findings, together with the potent anti-HIV-1 activity and favorable preclinical PK profile previously reported, position CL-197 as a promising candidate for the treatment of HIV-1 infection.

## Data Availability

The data generated during the current study are included in the article, and the raw data are available from the corresponding authors on reasonable request.

## SUPPLEMENTAL MATERIAL

Supplemental material aac.00984-26-s0001.docx Tables S1 to S5.

ASM does not own the copyrights to Supplemental Material that may be linked to, or accessed through, an article. The authors have granted ASM a non-exclusive, world-wide license to publish the Supplemental Material files. Please contact the corresponding author directly for reuse.
