# Supplementary material for: Safety and pharmacokinetics of CL-197, a potent compound with low cytotoxicity against HIV-1: a randomized, double-blind, placebo-controlled, first-in-human phase 1 clinical trial
Source: Antimicrob Agents Chemother. 2026 Sep 4;70(10):e00984-26. doi: 10.1128/aac.00984-26 (PMC13689352; doi:10.1128/aac.00984-26)
Supplement: Supplementary file 1 [file aac.00984-26-s0001.docx]

Supplemental Table 1. Top 10 AEs occurring in each group

| Adverse events | Occurrence frequency (%) | 1mg(n/N,%) | 10mg(n/N,%) | 30mg(n/N,%) | 60mg(n/N,%) | 100mg(n/N,%) |
| --- | --- | --- | --- | --- | --- | --- |
| sinus rhythm arrhythmia | 50.0% | 3/4,75% | 4/8,50% | 2/8,25% | 4/8,50% | 5/8,62.50% |
| decreased apolipoprotein B | 27.8.0% | -- | 1/8,12.5% | 5/8,62.50% | 4/8,50% | -- |
| decreased blood iron | 25.0% | 1/4,25% | 1/8,12.5% | 2/8,25% | 2/8,25% | 3/8,37.50% |
| elevated low-density lipoprotein | 19.4% | 1/4,25% | 1/8.12.5% | -- | 2/8,25% | 3/8,37.5% |
| increased triglycerides | 19.4% | 3/4,75% | -- | 1/8.12.5% | 2/8,25% | 1/8,12.50% |
| abnormal T-wave on electrocardiogram | 16.7% | -- | 4/8,50% | 1/8,12.5% | -- | 1/8,12.5% |
| sinus bradycardia | 16.7% | 1/4,25% | 1/8,12.50% | 3/8,37.5% | -- | 1/8,12.50% |
| elevated lipoprotein(a) | 13.9% | 2/4,50% | -- | 1/8,12.5% | -- | 2/8,25% |
| elevated blood lactate | 13.9% | 2/4,50% | -- | -- | 2/8,25% | 1/8,12.5 |
| elevated apolipoprotein B | 11.1% | -- | -- | -- | 2/8,25% | 2/8,25% |

Supplemental Table 2. Summary of PK parameters (Mean ± SD, CV%)

|  | 1mg (N=4) | 10mg (N=8) | 30mg (N=8) | 60mg (N=8) | 100mg (N=8) |
| --- | --- | --- | --- | --- | --- |
| C_max_（ng/mL） | 8.80±1.663  (18.9) | 102.15±31.472  (30.8) | 303.13±92.303  (30.5) | 577.13±102.754  (17.8) | 1204.13±257.136  (21.4) |
| AUC_0-t_（h*ng/mL） | 12.59±5.444  (43.2) | 231.83±34.657  (14.9) | 901.82±165.390  (18.3) | 1975.79±264.964  (13.4) | 3223.80±452.931  (14.0) |
| AUC_0-∞_（h*ng/mL） | 23.76±NE  (NE) | 253.73±36.954  (14.6) | 939.19±164.491  (17.5) | 2034.15±269.528  (13.3) | 3309.84±479.250  (14.5) |
| T_max_（h） | 1.00  (0.50~1.50) | 0.50  (0.50~1.00) | 1.00  (0.50~2.00) | 1.00  (0.50~2.00) | 0.50  (0.50~1.50) |
| t_1/2_（h） | 1.39  (1.39~1.39) | 2.20  (1.93~2.65) | 4.10  (2.89~7.01) | 5.61  (3.78~13.38) | 10.20  (3.35~15.40) |
| ke（/h） | 0.50±NE  (NE) | 0.32±0.033  (10.5) | 0.18±0.053  (30.2) | 0.13±0.042  (32.5) | 0.10±0.065  (63.1) |
| Vd（L） | 84.16±NE  (NE) | 127.46±20.665  (16.2) | 201.31±57.869  (28.7) | 258.93±97.099  (37.5) | 410.43±208.813  (50.9) |
| CL（L/h） | 42.09±NE  (NE) | 40.16±5.907  (14.7) | 33.05±7.298  (22.1) | 29.93±3.746  (12.5) | 30.73±4.069  (13.2) |
| MRT_0-t_（h） | 1.18±0.290  (24.6) | 2.57±0.216  (8.4) | 4.04±0.800  (19.8) | 4.74±0.865  (18.3) | 4.65±1.215  (26.1) |
| MRT_0-∞_（h） | 2.29±NE  (NE) | 3.32±0.391  (11.8) | 4.88±0.937  (19.2) | 5.66±1.286  (22.7) | 5.92±1.990  (33.6) |

Note: Tmax and t_1/2_ are presented as median (minimum, maximum)

Supplemental Table 3. Validation of linear PK characteristics for CL-197 in plasma via confidence interval approach

| Pharmacokinetic paramete | Linear regression equation | Slope(β) of linear regression equation | 90% Confidence interval of β | Jugement interval |
| --- | --- | --- | --- | --- |
| lnAUC_0-t_(h*ng/mL) | lnAUC_0-t_=1.22lnDose+2.56 | 1.22 | [1.18~1.26] | [0.95~1.05] |
| lnAUC_0-∞_(h*ng/mL) | lnAUC_0-∞_=1.11lnDose+3.04 | 1.11 | [1.06~1.15] | [0.95~1.05] |
| lnC_max_(ng/mL) | lnC_max_=1.05lnDose+2.14 | 1.05 | [1.0~1.1] | [0.92~1.08] |

Supplemental Table 4. Summary of PK parameters for CL-197-TP in PBMCs of subjects (Mean ± SD, CV%)

| Pharmacokinetic paramete | 30mg (N=8) | 60mg (N=8) |
| --- | --- | --- |
| C_max_(ng/10^6^ cell) | 1.34±1.255(93.8) | 2.67±0.835(31.3) |
| AUC_0-t_(h*ng/10^6^ cell) | 80.66±58.856(73.0) | 165.57±23.000(13.9) |
| AUC_0-∞_(h*ng/10^6^ cell) | 89.48±59.403(66.4) | 176.20±26.420(15.0) |
| T_max_(h) | 12.00(8.00~24.00) | 12.00(4.00~24.00) |
| t_1/2_(h) | 61.29(19.40~78.67) | 43.80(39.65~99.94) |
| K_e_(/h) | 0.02±0.011(65.3) | 0.02±0.005(31.8) |
| Vd(10^6^ cell) | 37.16±25.964(69.9) | 26.66±8.773(32.9) |
| CL(10^6^ cell/h) | 0.46±0.246(53.1) | 0.35±0.063(18.1) |
| MRT_0-t_(h) | 54.17±11.459(21.2) | 46.87±7.659(16.3) |
| MRT_0-∞_(h) | 79.39±24.884(31.3) | 60.88±11.681(19.2) |
| Note: Tmax and t_1/2_ are presented as median (minimum, maximum) | | |

Supplemental Table 5. Report of cumulative urinary excretion and fraction excreted unchanged in subjects during sensitivity analysis (Mean ± SD, CV%)

|  | 1mg (N=4) | 10mg (N=8) | 30mg (N=8) | 60mg (N=8) | 100mg (N=8) |
| --- | --- | --- | --- | --- | --- |
| Ae_0-72_(ug) | 587.44±64.012(10.9) | 6388.03±1101.205(17.2) | 20910.74±16879.759(80.7) | 36758.92±14511.940(39.5) | 65498.38±11595.769(17.7) |
| Fe_0-72_(%) | 58.74±6.399(10.9) | 63.88±11.013(17.2) | 69.70±56.265(80.7) | 61.26±24.186(39.5) | 65.50±11.596(17.7) |
|  | | | | | |
|  | | | | | |
